# 肺癌患者围手术期振动正压呼气训练有助于加速康复吗？

**DOI:** 10.3779/j.issn.1009-3419.2018.12.06

**Published:** 2018-12-20

**Authors:** 鹏飞 李, 玉田 赖, 坤 周, 建华 苏, 国卫 车

**Affiliations:** 1 610041 成都，四川大学华西医院胸外科 Department of Thoracic Surgery, West China Hospital, Sichuan University, Chengdu 610041, China; 2 610041 成都，四川大学华西医院康复科 Department of Rehabilitation, West China Hospital, Sichuan University, Chengdu 610041, China

**Keywords:** 振动正压呼气训练, 正压呼气装置, 肺肿瘤, 胸腔镜肺叶切除术, Oscillatory positive expiratory pressure, Acapella, Lung neoplasms, Video-assisted thoracic surgery

## Abstract

**背景与目的:**

振动正压呼气（oscillatory positive expiratory pressure, OPEP）训练是一种通过正压呼气装置（acapella）进行的呼吸训练。OPEP在慢性阻塞性肺疾病、支气管扩张症、肺囊肿等疾病的临床价值已经得到广泛探讨，但其在肺癌手术患者围术期的应用价值尚有待探索。本研究旨在探索围术期进行振动正压呼气训练对胸腔镜肺癌患者术后并发症发生率、肺功能、生活质量的影响。

**方法:**

前瞻性收集2017年9月15日-2018年1月15日四川大学华西医院胸外科单个医疗组行胸腔镜肺叶切除的原发性非小细胞癌患者69例，随机分成实验组（35例）和对照组（34例）。实验组（acapella group, AG）围手术期采用振动正压呼气训练，对照组（control group, CG）进行常规围术期处理。对比分析两组在术后并发症发生率、肺功能、生活质量方面的差异。

**结果:**

术后肺部并发症和肺不张在AG（2.9%, 0.0%）显著低于CG（20.6%, 14.7%）（*P*=0.03, *P*=0.03）；平均住院日和术后住院日在AG（10.86±5.64, 5.09±4.55）d显著短于CG（14.41±4.58, 7.59±3.21）d（*P*=0.01, *P*=0.01）；住院药物费用在AG（4, 413.60±1, 772.35）￥显著低于CG（6, 490.35±3, 367.66）￥（*P*=0.01）。出院当日第1秒用力呼气容积（forced expiratory volume in the first second, FEV_1_）和呼气峰流速（peak expiratory flow, PEF）在AG[(1.50±0.32) L, (252.06±75.27) L/min]显著高于CG[(1.34±0.19) L, (216.94±49.72) L/min]（*P*=0.03, *P*=0.03）。

**结论:**

肺癌患者围手术期使用振动正压呼气训练有助于降低肺部并发症，同时能够加速患者康复。

## Abstract (en)

**Background and objective:**

Oscillatory positive expiratory pressure (OPEP) training is a kind of breathing exercise with Acapella. The clinical value of OPEP has been widely discussed in chronic obstructive pulmonary disease, bronchiectasis as well as pulmonary cyst. However, few studies have explored the application of OPEP in surgery lung cancer patients underwent lobectomy. Thus, the aim of this study is to explore the impact of the application of OPEP device (acapella) in lung cancer patients undergoing video-assisted thorascopic surgery (VATS).

**Methods:**

Sixty-nine patients receiving VATS lobectomy in Department of Thoracic Surgery, West China Hospital, Sichuan University from September 15, 2017 to January 15, 2018 were randomly divided into the acapella group (AG) or the control group (CG). The patients in the AG received oscillating positive expiratory pressure training and the CG underwent standard perioperative treatment. The differences of morbidity, pulmonary function, quality of life were compared between the two groups.

**Results:**

Thirty-five patients were assigned to the AG and thirty-four patients were assigned to the CG. The incidences of postoperative pulmonary complications (PPCs) and atelectasis (2.9%, 0.0%) in the AG were significantly lower than that in the CG (20.6%, 14.7%)(*P*=0.03, *P*=0.03). The duration of total hospital stay and postoperative hospital stay in the AG (10.86±5.64, 5.09±4.55) d were significantly shorter than that in the CG (10.86±5.64, 5.09±4.55) d (*P*=0.01, *P*=0.01). The drug cost in the AG (4, 413.60±1, 772.35) ￥ were significantly lower than that in the CG (6, 490.35±3, 367.66) ￥ (*P*=0.01). The patients in the AG had better forced expiratory volume in the first second and peak expiratory flow [(1.50±0.32) L, (252.06±75.27) L/min] compared with the CG [(1.34±0.19) L, (216.94±49.72) L/min] (*P*=0.03, *P*=0.03) at discharge.

**Conclusion:**

The application of OPEP device during the perioperative period was valuable in decreasing PPCs and enhancing recovery for lung cancer patients receiving VATS lobectomy.

外科手术是早期非小细胞肺癌（non-small cell lung cancer, NSCLC）患者首选的治疗方式，随着肺癌筛查的普及和低剂量螺旋计算机断层扫描（computed tomography, CT）的使用，越来越多的肺癌患者得以早期发现并得到及时有效的手术治疗^[1, 2]^。近年来外科手术技术有了很大改进，但术后肺部并发症（postoperative pulmonary complication, PPC），尤其是痰潴留及由此导致的肺部感染等并发症的发生率在2%-40%^[3]^。术后并发症不但影响患者肺功能的恢复，也延长术后住院日，增加住院费用。振动正压呼气（oscillatory positive expiratory pressure, OPEP）训练是一种通过正压呼气装置（acapella）进行的呼吸训练。其作用在于，一方面使用者在呼气时遇到阻力产生肺内正压，进而撑开气道；另一方面，呼气产生的气流促进肺内分泌物离开肺泡表面，推动上行至上段气管，最终通过咳嗽将分泌物咳出^[4-6]^。振动正压呼气训练虽在支气管扩张症、肺囊性纤维化等疾病中的祛痰作用得到广泛认可^[7-10]^，但其在外科病人围手术期应用的相关研究较少，其适用性和有效性还有待证实。本研究旨在探索围术期振动正压呼气在降低肺癌患者术后PPC发生率、提高患者肺功能和生活质量及加速康复中的应用价值。

## 1 资料与方法

### 1.1 研究对象

前瞻性收集四川大学华西医院胸外科2017年9月15日-2018年1月15日华西医学院胸外科单个医疗组行肺叶切除的肺癌患者，随机分为实验组（acapella group, AG）与对照组（control group, CG），CG行常规围手术期处理，包括入院宣教与术前宣教、腹式呼吸训练、咳嗽训练、围术期疼痛管理等一系列内容。AG在常规围术期处理的基础上，进行振动正压呼气训练。纳入标准：①病理确诊为NSCLC；②手术方式为单向式胸腔镜（video-assisted thorascopic surgery, VATS）肺叶切除+系统性淋巴结清扫；③年龄45岁-75岁；④签署知情同意书。排除标准：①术前1个月有激素应用史；②近期罹患心梗、心衰、神经肌肉类疾病；③患者拒绝或不能耐受呼吸训练；④其他呼吸训练相关禁忌，如活动性咯血；⑤血流动力学不稳定；⑥未治疗的气胸患者；⑦接受过放化疗；⑧开胸手术或胸腔镜手术中转开胸患者；⑨手术时间超过4 h、术中出血 > 1, 000 mL等；⑩既往有胸部手术病史；最终共入组69例，平均年龄（57.26±9.19）岁，男性32例，女性37岁，有吸烟史者21例（30.4%）；AG 35例，CG 34例，两组之间在年龄、性别、体质指数、吸烟史、合并疾病等方面无差异（*P* > 0.05），见表 1。

**1:** 两组患者临床资料 Characteristics of the patients

Characteristics	Total (*n*=69)	AG (*n*=35)	CG (*n*=34)	*P*
Age (Mean±SD, yr)	57.26±9.19	58.03±9.56	56.47±8.86	0.49
Gender (Female)	37 (53.6%)	18 (51.4%)	17 (48.6%)	0.81
BMI (Mean±SD, kg/m^2^)	23.34±2.82	23.02±2.68	23.67±2.96	0.35
Smoking history				> 0.99
Current or former	21 (30.4%)	11 (31.4%)	10 (29.4%)	
Never	48 (69.6%)	24 (68.6%)	24 (70.6%)	
ASA score				0.79
2	49 (71.0%)	24 (68.6%)	25 (73.5%)	
3	20 (29.0%)	11 (31.4%)	9 (26.5%)	
Pulmonary function (Mean±SD)				
FEV_1_ (L)		2.40±0.44	2.43±0.43	0.73
PEF (L/min)		410.17±82.77	404.38±48.02	0.59
Quality of life (Mean±SD)				
Global QoL		75.48±12.93	75.25±9.73	0.93
Physical function		90.67±7.79	93.33±5.68	0.11
Social function		80.48±13.09	77.94±8.91	0.35
Dyspnoea score		10.48±15.70	11.76±16.17	0.74
Fatigue score		10.79±10.26	10.13±6.90	0.75
Perioperative morbidity				
Hypertension	17 (24.6%)	9 (25.7%)	8 (23.5%)	> 0.99
COPD	4 (5.8%)	4 (11.4%)	0	0.11
Diabetes	2 (2.9%)	2 (5.7%)	0	0.49
CHD	1 (1.4%)	0	1 (2.9%)	0.49
Pathological type				> 0.99
Adeno	67 (97.1%)	34 (97.1%)	33 (97.1%)	
SCC	2 (2.9%)	1 (2.9%)	1 (2.9%)	
BMI: body mass index; ASA: American Society of Anesthesiologists; QoL: quality of life; COPD: chronic obstructive pulmonary disease; CHD: coronary heart disease; FEV_1_: forced expiratory volume in one second; PEF: peak expiratory flow; Adeno: adenocarcinoma; SCC: squamous cell carcinoma.				

### 1.2 随机化方法

采用随机数字表法对患者进行随机分组，患者入组后由两名实验人员向患者宣教训练方法，每天两次巡视患者，观察并记录患者的训练情况。

### 1.3 实验伦理

本实验已通过四川大学华西医院临床试验与生物医学专委会伦理（2017-403）审核，并在世界卫生组织国际临床试验注册平台注册，注册号为ChiCTR1800014512。

### 1.4 实验流程

#### 1.4.1 入院评估内容

入院第1天：对所有入组患者进行评估，评估内容包括：肺功能、健康相关生活质量（health-related quality of life：HRQoL，采用EORTC QLQ-C30量表）评估^[11]^。完成评估后，随机分成AG和CG。

#### 1.4.2 训练方法

AG使用振动正压呼气训练装置（Acapella Choice, Smith Medical ASD公司USA，购置于四川天乐康科技有限公司）进行训练。训练步骤：Ⅰ：调整阻力表盘将阻力设为最低，待患者适应后设为三档；Ⅱ：患者吸气，吸气量应稍大于正常潮气量；Ⅲ：紧闭双唇完全包绕咬嘴；Ⅳ：屏气2 s-3 s；Ⅴ：紧闭双唇完全包绕咬嘴，持续呼气3 s-4 s（如缓慢吹蜡烛），完成一次正压呼吸。每组进行10次-20次呼吸训练，每组完成后取下呼吸训练装置，进行2次-3次用力哈气或咳嗽，患者在清醒状态下每隔2 h进行一组训练。

### 1.5 观察指标

#### 1.5.1 术后30 d内PPC发生率

PPC包括：①术后肺炎（postoperative pneumonia, POP）；②肺叶或全肺肺不张，需行纤支镜吸痰；③肺栓塞；④呼吸衰竭或急性呼吸窘迫症（acute respiratory distress syndrome, ARDS）：术后持续通气支持 > 24 h或需要重新插管；⑤气管拔管后重新插管；⑥术后早期机械通气 > 48 h；⑦气管切开等。肺部并发症的诊断标准参照美国胸外科医师协会（Society of Thoracic Surgeons, STS）和欧洲胸科医师协会（European Society of Thoracic Surgeons, ESTS）对胸外科术后并发症的联合定义^[11]^。

#### 1.5.2 第1秒用力呼气容积（forced expiratory volume in the first second, FEV_1_）

肺功能检测的常用指标。

#### 1.5.3 呼气峰流速（peak expiratory flow, PEF）

反映气道通畅性和呼吸肌力量的指标，可衡量咳嗽咳痰的效率^[12]^。

#### 1.5.4 生活质量量表评分（EORTC QLQ-C30 & LC13_CN, version 3）

该量表包括C-30与其肺癌子模块LC13（lung cancer 13）共同组成，包括社会、躯体、情感、角色和认知5个维度^[13]^。

#### 1.5.5 平均住院日

入院当天到出院当天（入院当天不算在内，出院当天算在内）。

#### 1.5.6 术后住院日

手术后第1天到出院当天时间（出院当天计算在内）。

#### 1.5.7 平均住院费用

住院期间所产生的费用，不包括门诊检查或治疗所产生的费用。

#### 1.5.8 平均住院药物费用

包括住院期间使用的所有药物的费用。

#### 1.5.9 抗生素使用时间

手术当天到停用抗生素。

### 1.6 统计学方法

应用SPSS 22.0软件分析结果，计数资料用实际例数及百分比表示，组间比较采用独立样本的卡方检验或*Fisher*确切概率法，计量资料采用均数±标准差（Mean±SD）表示，组间比较采用*t*检验，以*P* < 0.05为差异有统计学意义。

## 2 结果

### 2.1 围手术期OPEP降低肺部相关并发症

术后肺部并发症15例（21.7%, 15/69），其中PPCs 8例（11.6%, 8/69）、肺不张5例（7.2%, 5/69），术后肺炎对照组1例（1.45%, 1/69）。术后PPCs和肺不张在AG（2.9%, 0）显著低于CG（20.6%, 14.7%）（*P*=0.03, *P*=0.01）。其他常见并发症包括术后持续性肺漏气（10.1%, 7/69）和皮下气肿（8.7%, 6/69）；术后持续性肺漏气和皮下气肿在AG（11.4%, 8.6%）与CG（8.8%, 8.8%）无统计学差异（*P* > 0.99, *P* > 0.99），见表 2。

**2:** 两组并发症分析 Complications classified by two groups

Complications	AG	CG	*P*
Total complications	6 (17.1%)	9 (26.5%)	0.39
PPCs	1 (2.9%)	7 (20.6%)	0.03
POP	0	1 (2.9%)	0.49
Atelectasis	0	5 (14.7%)	0.03
Persistent air leak	4 (11.4%)	3 (8.8%)	1.00
Subcutaneous emphysema	3 (8.6%)	3 (8.8%)	1.00
Initial ventilator support > 48 h	0	1 (2.9%)	0.49
Hoarseness	1 (2.9%)	0	1.00
PPCs: postoperative pulmonary complications; POP: postoperative pneumonia.			

### 2.2 围手术期OPEP提高肺功能和生活质量

患者出院当天FEV_1_（1.50±0.32）L和PEF（252.06±75.27）L/min在实验组均显著高于对照组（1.34±0.19）L、（216.94±49.72）L/min，（*P*=0.03, *P*=0.03）。出院当天两组患者总体生活质量评分、躯体功能、社会功能、呼吸困难分和疲劳评分在AG均优于CG组，但无统计学意义，见表 3。

**3:** 两组患者住院日、住院花费、肺功能及生活治疗的比较（Mean±SD） Difference of hospital stay, in-hospital expense and pulmonary function between the two groups (Mean±SD)

Variables	AG (*n*=35)	CG (*n*=34)	*P*
Length of stay			
Total (d)	10.86±5.64	14.41±4.58	0.01
Preoperative (d)	5.77±1.93	6.53±2.39	0.15
Postoperative (d)	5.09±4.55	7.59±3.21	0.01
Hospital expense			
Total cost (¥)	48, 581.80±9, 966.73	47, 093.38±7, 830.94	0.49
Drug cost (¥)	4, 413.60±1, 772.35	6, 490.35±3, 367.66	0.00
Duration of antibiotic use (d)	2.06±1.64	2.32±0.95	0.41
Duration of chest tube (d)	3.09±3.79	4.12±3.11	0.22
Pulmonary function			
FEV_1_ (the day before operation, L)	2.39±0.38	2.50±0.42	0.25
PEF (the day before operation, L/min)	417.31±83.02	407.21±47.13	0.54
FEV_1_ (at discharge, L)	1.50±0.32	1.34±0.19	0.03
PEF (at discharge, L)	252.06±75.27	216.94±49.72	0.03
QoL evaluation (the day before operation)			
Global QoL^*^	68.63±11.08	65.44±10.08	0.22
Physical function^*^	88.19±3.65	86.86±3.06	0.11
Social function^*^	80.95±11.53	81.86±1.14	0.74
Dyspnoea score^†^	20.00±16.57	22.58±18.84	0.52
Fatigue score^†^	10.42±8.44	10.04±7.23	0.85
QoL evaluation (the day discharged)			
Global QoL^*^	65.47±11.28	62.50±7.18	0.20
Physical function^*^	87.43±3.53	86.86±3.06	0.48
Social function^*^	80.00±11.99	79.90±12.83	0.94
Dyspnoea score^†^	20.00±16.57	23.53±15.42	0.36
Fatigue score^†^	11.11±8.52	11.44±8..42	0.87
^*^Higher scores indicate better functioning (scaled from 0-100); ^†^Lower scores indicate less dyspnea (scaled from 0-100).			

### 2.3 围手术期OPEP缩短住院时间和降低住院费用

平均住院日和术后住院日在AG（10.86±5.64, 5.09±4.55）d均显著短于CG（14.41±4.58, 7.59±3.21）d（*P*=0.01, *P*=0.01）。平均住院费在AG（48, 581.80±9, 966.73）¥与CG（47, 093.38±7, 830.94）¥无统计学差异（*P*=0.49）；而平均住院药费在AG（4, 413.60±1, 772.35）¥显著低于CG（6, 490.35±3, 367.66）¥（*P* < 0.001），见表 3。

## 3 讨论

术后肺炎和肺不张是胸外科患者术后常见的并发症，其原因可能在于胸外科手术导致的术后疼痛和限制性通气障碍影响了患者的自主有效地咳嗽、排痰。目前多通过以下两种途径辅助患者排痰：一是化痰、祛痰药物，此类药物可通过静脉、雾化吸入、口服等途径给药，但药物的使用不可避免地增加患者的医疗花费，且某些祛痰药还会带来恶心、呕吐、过敏、支气管痉挛等不良反应；另一途径为物理祛痰途径，包括人工或机器辅助振动拍背、压迫气管诱导咳嗽等，但这一类方法祛痰效果并不确切，且可能引起患者不适。振动正压呼气训练是通过气流的振动使粘滞的分泌物松动，并通过呼气时产生的正压扩张气道，通过呼吸道粘膜纤毛的摆动将松动的分泌物自下而上推入上部的气道，更有利于通过咳嗽等将分泌物排出。振动正压呼气训练已经在支气管扩张症、慢性阻塞性肺疾病、肺囊肿等痰液增多的内科性疾病中得到广泛的应用，近年来其在围手术期的使用也有研究进行了初步探索^[5, 6]^，但在肺外科领域的研究较少。基于此，我们实施此项临床试验，以进一步探索振动正压呼气训练在肺癌患者围手术期使用的安全性和有效性。

本研究共纳入69例行胸腔镜肺叶切除的肺癌患者，两组均未出现死亡病例。两组虽在术后总体并发症发生率方面无明显统计学差异，但Acapella组术后肺部并发症发生率明显低于对照组，术后肺不张发生率也低于对照组。这表明对于胸腔镜肺癌手术患者，围手术期使用Acapella能够有效帮助患者排痰，促进早期肺复张，减少术后肺不张，从而降低肺部感染、呼吸衰竭、重新气管插管等并发症的发生率。

作为肺功能检查的一项常用指标，有研究^[12]^表明PEF在一定程度上能够反映患者咳嗽咳痰的能力，且术前PEF与术后发生肺部相关并发症有密切联系。本研究中，出院当天Acapella组患者PEF显著高于对照组，说明围手术期使用Acapella能够在一定程度上提高患者的排痰能力。一项肺部手术后患者使用Acapella的研究^[5]^表明，术后给予振动正压呼气训练能够增加患者的排痰量，使患者术后排浓稠痰的时间减少，术后排痰量更多，痰液更容易咳出。另一项对比Acapella与激励式肺量计的研究表明，肺癌患者术后使用Acapella能够更有效地促进患者排除呼吸道分泌物，且Acapella舒适度更高^[6]^。我们的研究虽没有关注患者术后排痰量和性状的变化，但与术后排痰密切相关的并发症肺不张在Acapella组明显低于对照组，可以从侧面反映患者的排痰能力。与上述两项研究相比，我们对于进入Acapella组的患者术前便开始进行正压呼气训练，这一方面能帮助患者在术后更有效地掌握Acapella的使用方法；另一方面对于长期吸烟和合并COPD等痰液量增多的患者在术前就进行干预，降低患者术后并发症的风险，同时术前一天肺功能检测结果表明（表 3），术前使用Acapella在一定程度上可提高患者肺功能。

本研究结果还显示，Acapella组术后住院日、平均住院日、药物花费、抗生素使用时间明显低于对照组，表明肺癌患者围手术期使用Acapella是有效的。有研究^[14]^表明对合并术后并发症危险因素的患者术前进行综合肺康复训练可有效降低患者术后并发症，缩短术后住院时间，加速患者康复。我们思考，作为一种简便易操作的排痰训练装置，可以将围术期振动正压呼吸训练加入肺癌患者肺康复训练方案。尤其对于合并有吸烟史、年龄大、合并COPD、低肺功能等术后并发症高危因素的患者^[15, 16]^，围手术期使用Acapella的意义可能更大。

本研究还存在如下不足：①研究纳入单个医疗中心单个医疗组的的患者，样本含量较小，可能存在选择偏倚；②本研究纳入患者年龄相对年轻，多数患者因体检发现肺部病变入院，这与肺癌筛查的普及和越来越多的早期肺癌患者及时发现有关，这也是两组患者的生活质量评分无统计学差异的一个原因；③本组实验纳入胸腔镜肺叶切除的肺癌患者，对于早期发现的肺癌患者，亚肺叶切除越来越受到胸外科医师的重视，振动正压呼气训练对于此部分患者是否有效，尚有待探索，这也是我们下一步的研究方向。因此，未来需要更大样本含量的多中心研究来证实我们的结论。

总之，胸腔镜肺癌手术患者围手术期使用振动正压呼气训练能够降低患者术后并发症、加速患者康复，可作为肺癌患者围术期的一项干预措施。
